# Supplementary material for: Development and evaluation of three automated media pooling and molecular diagnostic systems for the detection of SARS-CoV-2
Source: Microbiol Spectr. 2024 Jan 30;12(3):e03684-23. doi: 10.1128/spectrum.03684-23 (PMC10913432; doi:10.1128/spectrum.03684-23)
Supplement: Tables S1 to S4, Figure S1 — Supplemental tables and figure. [file spectrum.03684-23-s0001.docx]

**Development and evaluation of three automated media pooling and molecular diagnostic systems for the detection of SARS-CoV-2**

**Supplemental Materials**

**Table S1. Data used for the analytical sensitivity analysis.**

| Pooling system (specimen) | Size of pool | Viral copies/sample mL, positive rate (no. of replicates, positive/tested) | | | | | | | | | |
| --- | --- | --- | --- | --- | --- | --- | --- | --- | --- | --- | --- |
|  |  | 25,000 | 10,000 | 5,000 | 2,500 | 1,000 | 500 | 250 | 100 | 75 | 50 |
| geneLEAD-PS (saliva) | Individual testing | ND | 100% (6/6) | 100% (6/6) | 100% (6/6) | 100% (6/6) | 100% (6/6) | 100% (10/10) | 100% (20/20) | 60% (6/10) | 17% (2/12) |
|  | 4 | ND | 100% (6/6) | 100% (6/6) | 100% (6/6) | 100% (6/6) | 95% (19/20) | 70% (7/10) | 33% (2/6) | ND | ND |
|  | 6 | ND | 100% (6/6) | 100% (6/6) | 100% (6/6) | 100% (20/20) | 79% (11/14) | 57% (8/14) | 33% (2/6) | ND | ND |
|  | 8 | ND | 100% (6/6) | 100% (6/6) | 100% (6/6) | 95% (19/20) | 79% (11/14) | 33% (2/6) | 0% (0/6) | ND | ND |
| Panther-PS (saliva) | Individual testing | ND | 100% (16/16) | 100% (16/16) | 100% (16/16) | 94% (15/16) | 44% (7/16) | 60% (9/15) | 0% (0/16) | ND | ND |
|  | 4 | ND | 100% (6/6) | 100% (6/6) | 100% (6/6) | 33% (2/6) | 33% (2/6) | 0% (0/6) | 0% (0/6) | ND | ND |
|  | 6 | 100% (7/7) | 100% (6/6) | 83% (5/6) | 83% (5/6) | 17% (1/6) | 0% (0/6) | 0% (0/6) | 0% (0/6) | ND | ND |
|  | 10 | 100% (7/7) | 100% (6/6) | 33% (2/6) | 33% (2/6) | 17% (1/6) | 0% (0/6) | 0% (0/6) | 0% (0/6) | ND | ND |
| Panther-PS (nasal swab; Aptima®) | Individual testing | ND | 100% (13/13) | 100% (13/13) | 100% (13/13) | 100% (20/20) | 95% (19/20) | 77% (10/13) | 38% (5/13) | ND | ND |
|  | 4 | ND | 100% (10/10) | 100% (10/10) | 100% (10/10) | 90% (9/10) | 70% (7/10) | 0% (0/10) | 0% (0/10) | ND | ND |
|  | 6 | ND | 100% (10/10) | 100% (10/10) | 90% (9/10) | 90% (9/10) | 30% (3/10) | 50% (5/10) | 10% (1/10) | ND | ND |
|  | 10 | ND | 100% (10/10) | 100% (10/10) | 90% (9/10) | 70% (7/10) | 40% (4/10) | 20% (2/10) | 0% (0/10) | ND | ND |
| Biomek-PS (saliva) | Individual testing | ND | 100% (9/9) | 100% (9/9) | 100% (9/9) | 100% (18/18) | 94% (17/18) | 89% (8/9) | 44% (4/9) | ND | ND |
|  | 4 | 100% (18/18) | 100% (18/18) | 100% (19/19) | 94% (17/18) | 56% (5/9) | 0% (0/9) | ND | ND | ND | ND |
|  | 6 | 100% (9/9) | 100% (9/9) | 100% (9/9) | 89% (8/9) | 11% (1/9) | 0% (0/9) | ND | ND | ND | ND |
|  | 10 | 100% (9/9) | 100% (9/9) | 100% (9/9) | 50% (9/18) | 22% (2/9) | 0% (0/9) | ND | ND | ND | ND |

ND, not determined.

**Table S2. Details of pooled testing in the validation study.**

| Pooling system (size of pool) | Specimen | Number of positive/negative samples tested (prevalence) | Number of pools tested, positive/negative results^a^ | Negative pool fraction (fraction of samples that were determined to be negative by only testing of pooled samples) | Reduction rate in number of tests^b^ (a total number of tests in pooling testing) | Retest rate^c^ (pooling/individual testing) | Invalid rate (pooling/individual testing) |
| --- | --- | --- | --- | --- | --- | --- | --- |
| geneLEAD-PS (6) | Saliva | 31/1,403 (2.2%) | 31/208 | 87.0% | 70.4% (425) | 0%/1.0% | 0%/0.5%^d^ |
|  | Nasopharyngeal swab | 21/527 (3.8%) | 14/78^e^ | 85.4% | 67.9% (176^e^) | 0%/4.8% | 0%/0% |
|  | Nasal swab | 17/449 (3.6%) | 9/69^e^ | 88.8% | 71.7% (132^e^) | 2.6%/3.8% | 0%/1.9%^d^ |
|  | All | 69/2,379 (2.8%) | 54/354^e^ | 86.8% | 70.1% (732^e^) | 0.5%/2.5% | 0%/0.6% |
| Panther-PS (4) | Nasal swab (Aptima®) | 45/3,183 (1.4%) | 39/768 | 95.2% | 70.2% (963) | 0%/2.6% | 0%/2.6%^f^ |
| Biomek-PS (4) | Saliva | 33/3,383 (1.0%) | 33/821 | 96.1% | 71.1% (986) | 0%/0% | 0%/0% |
|  | Nasopharyngeal swab | 26/1,271 (2.0%) | 23/303 | 93.8% | 68.1% (414) | 0%/0% | 0%/0% |
|  | Nasal swab | 47/1,660 (2.8%) | 40/388 | 90.7% | 65.3% (592) | 0%/0% | 0%/0% |
|  | All | 106/6,314 (1.7%) | 94/1511 | 94.1% | 69.1% (1985) | 0%/0% | 0%/0% |

^a^ Positive pools may contain multiple positive samples. The number of individual tests can be calculated by the number of pools with positive results multiplied by the size of the pool.

^b^ The reduction rate in the number of tests was calculated using the following formula: 1 - (the total number of tests needed in pooling testing [the number of pools tested plus the number of individual samples tested due to positive pools]) divided by the number of tests (samples) in individual testing.

^c^ In geneLEAD-PS, the retest rate is a sum of the retest rate due to the assay interpretation criteria (see Table S1) and the invalid rate. In Panther-PS and Biomek-PS, the retest rate is equal to the invalid rate.

^d^ One saliva sample and one nasal swab sample were judged to be invalid due to PCR error and interpretive criteria (the Ct value of extraction control >30), respectively. For both samples, test results could be obtained by retesting.

^e^ One pool consisted of 4 negative nasal swabs and 2 negative nasopharyngeal swabs, and the pool tested negative. The number of pools in each specimen did not add up to the number of pools for all specimens.

^f^ Results for four samples could not be obtained due to sample clots. For all four samples, the results could be obtained by retesting.

**Table S3. Assay interpretive criteria.**

| Assay (manufacturer or developer) | SARS-CoV-2 detection target | Internal control | Positive criteria | Negative criteria | Invalid criteria |
| --- | --- | --- | --- | --- | --- |
| LeaDEA VIASURE SARS-CoV-2 PCR Kit (Precision System Science) | Orf1ab and N genes separately | Extraction control (EC) | Ct value of either target ≤40, irrespective of Ct value of EC; if only one target was detected, the sample was retested and the second result was adopted | Ct value of either target >40 and Ct value of EC ≤30 | Ct value of EC >30 and positive criteria not met |
| Aptima SARS-CoV-2 (Hologic Japan) | Two targets within Orf1ab gene as one target | Internal control, (IC) | Presence of SARS-CoV-2 target and valid IC result | Absence of SARS-CoV-2 target and valid IC result | Invalid result of SARS-CoV-2 target or IC |
| N2 assay (the National Institute of Infectious Disease in Japan) | N gene | Not included | Ct value of N gene < 40 | Ct value of N gene ≥ 40 | Not defined |

Ct, cycle threshold determined by a real-time PCR instrument.

**Table S4. Specimens used in the analyses.**

| Analysis | Pooling system, specimen type (number of samples) | | |
| --- | --- | --- | --- |
|  | geneLEAD-PS | Panther-PS | Biomek-PS |
| Analytical sensitivity | Saliva | Saliva^a^ and Aptima® nasal swab | Saliva |
| Mock sample evaluation | Saliva (400) | Saliva^a^ (400) and Aptima® nasal swab (400) | Saliva (400) |
| Clinical validation | Nasopharyngeal swab (548), nasal swab (466), saliva (1,434) | Aptima® nasal swab (3,228) | Nasopharyngeal swab (1,297), nasal swab (1,707), saliva (3,416) |

^a^ For saliva testing, 1 mL was transferred to an Aptima® sample tube containing 2.9 mL of buffer.

**Figure S1. Ct value comparisons between pooled and individual testing in the clinical validation study.** Only test-positive pools with one positive sample by the individual testing are shown. The Ct values of the Orf1ab and N genes tested with geneLEAD-PS among 45 pools are shown in Panels A and B, respectively. The Ct values of the N2 gene tested with Biomek-PS among 82 pools are shown in Panel C. The assay of Panther-PS is based on the transcription-mediated amplification method, which is a qualitative method and does not produce Ct values. The gray area indicates 95% confidence intervals. The dashed line shows a reference line of y=x.
